# Morphological divergence and flow-induced phenotypic plasticity in a native fish from anthropogenically altered stream habitats

**DOI:** 10.1002/ece3.842

**Published:** 2013-10-25

**Authors:** Nathan R Franssen, Laura K Stewart, Jacob F Schaefer

**Affiliations:** 1 Department of Biology and Museum of Southwestern Biology, University of New Mexico167 Castetter Hall, Albuquerque, New Mexico 87131; 2 Department of Biological Sciences, University of Southern MississippiHattiesburg, Mississippi 39406

**Keywords:** Contemporary evolution, ecomorphology, phenotypic plasticity, reservoirs, trait diversification

## Abstract

Understanding population-level responses to human-induced changes to habitats can elucidate the evolutionary consequences of rapid habitat alteration. Reservoirs constructed on streams expose stream fishes to novel selective pressures in these habitats. Assessing the drivers of trait divergence facilitated by these habitats will help identify evolutionary and ecological consequences of reservoir habitats. We tested for morphological divergence in a stream fish that occupies both stream and reservoir habitats. To assess contributions of genetic-level differences and phenotypic plasticity induced by flow variation, we spawned and reared individuals from both habitats types in flow and no flow conditions. Body shape significantly and consistently diverged in reservoir habitats compared with streams; individuals from reservoirs were shallower bodied with smaller heads compared with individuals from streams. Significant population-level differences in morphology persisted in offspring but morphological variation compared with field-collected individuals was limited to the head region. Populations demonstrated dissimilar flow-induced phenotypic plasticity when reared under flow, but phenotypic plasticity in response to flow variation was an unlikely explanation for observed phenotypic divergence in the field. Our results, together with previous investigations, suggest the environmental conditions currently thought to drive morphological change in reservoirs (i.e., predation and flow regimes) may not be the sole drivers of phenotypic change.

## Introduction

Understanding how populations respond to widespread and rapid environmental change will be a first step in elucidating the evolutionary consequences of disturbed habitats. Habitats altered by humans may destine populations to extirpation (Barnosky et al. 2011), but they may also constrain future evolutionary adaptability by lowering genetic diversity (Myers and Knoll 2001) or modify phenotypic traits of populations that can mediate ecosystem-level dynamics (Palkovacs et al. 2011). Stream impoundments across the planet have severely altered aquatic ecosystems (Dynesius and Nilsson 1994; Nilsson et al. 2005; Downing et al. 2006). While impounded streams and their associated reservoirs generally have deleterious impacts on native aquatic organisms (Dudgeon et al. 2006; Fullerton et al. 2010), they are widespread, can be treated as replicated units, and impact a wide-range of taxa, making them a good system to assess population-level responses to human-altered habitats.

The standing bodies of water above dams have drastically different environmental conditions compared with natural streams and likely exert novel selective pressures on stream fishes not experienced during their evolutionary history (Baxter 1977). Atypical selective pressures in these new habitats are evidenced by changes to native stream fish communities (e.g., obligate stream fishes are usually extirpated from reservoirs, increased abundances of piscivorous fishes, Taylor et al. 2001; Gido et al. 2009). But in spite of these pressures, some stream fishes persist in reservoirs and recent investigations have suggested these novel habitats may drive rapid phenotypic divergence in resident populations (Haas et al. 2010; Franssen 2011; Franssen et al. 2013). A mechanistic understanding of the factors that contribute to phenotypic divergence in reservoir habitats will elucidate the potential evolutionary consequences of altered habitats.

Variation in fish morphologies across habitats with variable water velocities combined with tight linkages between morphology and performance (Gosline 1971; Alexander 1967; Schaefer et al. 1999; Langerhans 2008) may help predict how reservoir habitats may alter phenotypes of reservoir-resident fishes. Fishes in lotic habitats often have fusiform morphologies that reduce drag and facilitate sustained swimming, whereas shallower anterior/head regions and increased caudal areas in lentic waters facilitates faster burst speeds and increased maneuverability (Gosline 1971; Alexander 1967; Langerhans and DeWitt 2004; Langerhans 2009). Intra- and interspecific body shape variation investigated in reservoirs and nearby streams substantiated these general patterns (Haas et al. 2010; Franssen 2011; Franssen et al. 2013). However, these lentic–lotic–morphological relationships are not universal. Some fishes can exhibit the opposite pattern with more streamlined body shapes in natural lakes compared with streams (e.g., Hendry et al. 2002; McGuigan et al. 2003; Krabbenhoft et al. 2009). Hence, species-specific ecologies and standing genetic variation within populations will likely regulate how species respond to reservoir habitats (Franssen et al. 2013); yet, these contingencies make predicting species-specific responses to reservoir habitats difficult.

Observed phenotypic shifts in reservoir habitats are potentially due to phenotypic plasticity as environmentally induced variation is widespread (Schlichting and Pigliucci 1998; West-Eberhard 2003). Nonetheless, environmentally contingent phenotypes can become canalized, where the previous environmental stimulus is no longer required to produce the trait (Waddington 1942; Schmalhausen 1949; Debat and David 2001). Even plastic responses to reservoir habitats may then facilitate evolution of resident populations (Ghalambor et al. 2007; Pfennig et al. 2010). Indeed, fishes can demonstrate flow-induced phenotypic plasticity (Keeley et al. 2007; Pakkasmaa and Piironen 2001; Grünbaum et al. 2007), and given that some fishes are plastic in response to variable flow regimes, phenotypic plasticity is potentially responsible for a portion of the morphological divergence observed in reservoir habitats. Assessing morphological responses of fishes to reservoirs, regardless of whether phenotypic divergence is due to “genetic” or plastic contributions, will lend insight into the potential evolutionary consequences of impoundments. While several recent studies have assessed morphological changes of fishes in reservoir habitats (e.g., Haas et al. 2010; Franssen 2011; Franssen et al. 2013), the contribution of phenotypic plasticity to observed changes has not been thoroughly evaluated (but see Franssen 2011), and the ubiquitous nature of divergence in species previously investigated is not clear.

**Figure 1:**
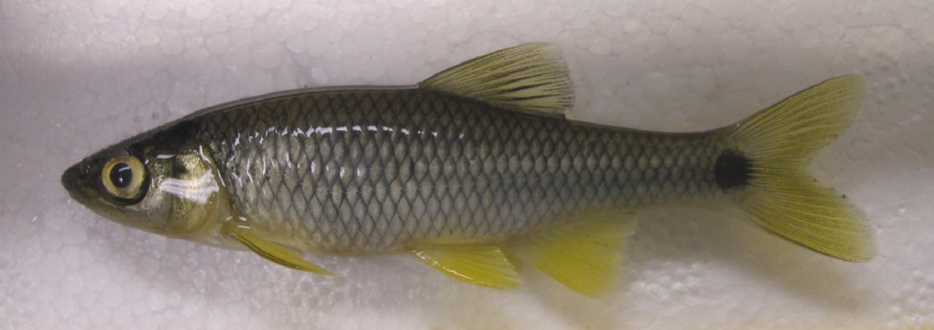
We assessed body shape variation in *Cyprinella venusta* from stream and reservoir habitats in northern Mississippi, USA. *Cyprinella venusta* is a small-bodied cyprinid that is relatively common in Gulf Coast stream systems.

Here, we tested for phenotypic divergence of *Cyprinella venusta*, a small-bodied native cyprinid, in reservoir habitats (Fig. 1). Although Haas et al. (2010) had previously demonstrated reservoir-induced morphological divergence in this species from the southeastern U.S.A (Mobile River Basin), we were interested in reproducing their results in the Mississippi River Basin to assess the repeatability of observed morphological responses to reservoirs. We also assessed the potential contribution of flow-induced phenotypic plasticity to observed morphological responses in the field by rearing offspring of a reservoir and a stream population in a common garden with lentic and flowing treatments. We predicted *C. venusta* in reservoir habitats would exhibit repeated trait shifts often associated with changes to flow variation (i.e., smaller anterior/head regions, deeper bodied with larger caudal areas). We also predicted *C. venusta* offspring reared in flowing water would have more fusiform body shapes compared with fish reared in lentic conditions and would parallel shape variation observed in reservoir and stream habitats.

## Materials and Methods

### Study sites and field collections

**Figure 2:**
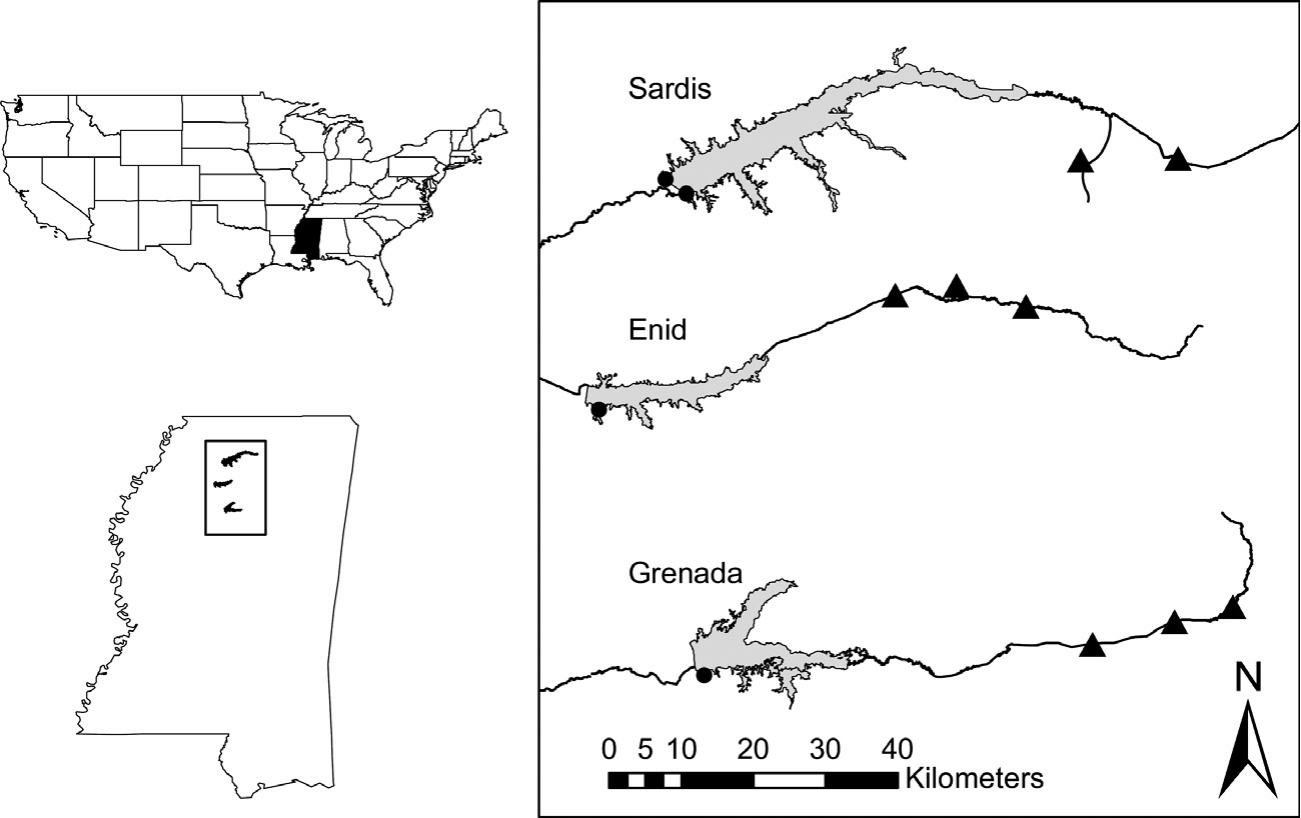
Map of sample sites where fish were collected to assess effects of reservoir habitats on morphological variation in *Cyprinella venusta*. Reservoir habitats are filled circles, and stream habitats are filled triangles.

We investigated shape variation in *C. venusta* from three reservoirs in the Hilly Gulf Coastal Plains in northwest Mississippi, USA (Fig. 2). Impoundment of the Little Tallahatchie River in 1940, the Yocona River in 1952, and the Yalobusha River in 1954 created Sardis, Enid, and Grenada Reservoirs, respectively. All three rivers historically flowed unimpounded into the Yazoo River in western Mississippi and the three basins contain similar fish faunas. *C. venusta* adults were collected between December 2011 and January 2012 from reservoir habitats by seine and a barge electrofisher, whereas stream habitats were only sampled with a seine. All sampled stream habitats were upstream of each reservoir with no known physical barrier obstructing migration between reservoir and stream habitats. Fish were euthanized on site with an overdose of MS-222, preserved and stored in 10% formalin and returned to the laboratory for data acquisition. One or two sites were sampled in each reservoir, and several stream sites were sampled in each basin but fish were opportunistically collected and individuals from sites within each basin and habitat (i.e., stream or reservoir) were combined (Table 1). The distance between reservoir and stream collections within each basin was at least 35 km (Euclidean distance).

**Table 1:** Sample sizes by basin and habitat and numbers and sizes of individuals reared in stream mesocosms

Study component	Basin	Habitat			

		Reservoir		Stream	
	
		*n*	SL (SD)	*n*	SL (SD)
Field collections	Enid	38	44.8 (7.5)	35	43.8 (5.8)
	Grenada	38	39.4 (6.6)	90	39.3 (5.0)
	Sardis	37	39.5 (7.7)	29	42.0 (5.7)

	Population	Treatment			
	
		Flow		No Flow	
		
		*n*	SL (SD)	*n*	SL (SD)
Mesocosm experiment	Reservoir	34	37.5 (5.7)	17	41.9 (8.0)
	Stream	31	41.2 (6.4)	25	38.0 (5.5)

### Morphological divergence and flow-induced plasticity

**Figure 3:**
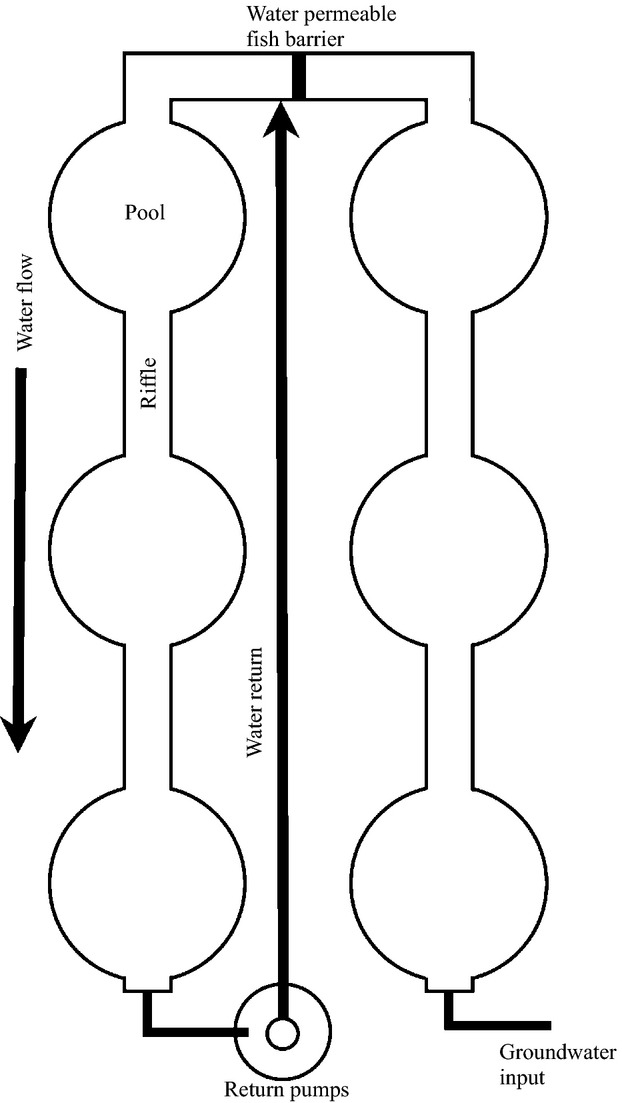
Aerial view of one of two mesocosms used to assess the relative contribution of population-of-origin and flow-induced phenotypic plasticity on shape variation of *Cyprinella venusta* offspring. The left side of each mesocosm was the flow treatment while the right side was the nonflow treatment.

We assessed potential genotypic differences and flow-induced phenotypic plasticity in morphology between reservoir and stream populations by spawning *C. venusta* adults from a reservoir and stream population and rearing their offspring in a common garden experiment with or without flow present. We collected adult *C. venusta* from Grenada Reservoir and the Yalobusha River upstream of the reservoir on February 2, 2012 and returned them to the laboratory. On 13 April 2012, 40 individuals (mean size = 51.9 mm standard length, range = 40.9–64.2 mm) from each population were split evenly (i.e., *n* = 20 selected randomly) and stocked into one of four experimental stream units (Matthews et al. 2006) located at the Lake Thoreau Environmental Center near Hattiesburg, MS (Fig. 3). Each unit consisted of three pools 183 cm diameter and three shallow riffle habitats (183 cm in length, see Matthews 2006). We applied flow in two of the four stream units (water velocity in riffle habitats ranged from 0.18 to 0.20 m/s) by use of four recirculating pumps for each unit (Danner MAG-Drive model 1800; Danner Manufacturing, Islandia, New York, discharge of 113 L min^−1^ per pump) that transferred water from the outflow end of units to the upstream riffle (Fig. 3). Pools and riffles had sand and gravel substrate (mined from local streams) and are colonized by various invertebrates that provide a natural diet that was not supplemented. Mesocosms were under 55% shade cloth and experienced a natural photo and thermal regime. Water quality was maintained by a constant supply (approximately 25 L h^−1^) of groundwater. Thus, we had a 2 × 2 factorial design with population crossed with flow and nonflow treatments. Adult *C. venusta* were allowed to spawn and then removed once juvenile fish were observed. All adults were removed by 3 July 2012. Experimental stream units were then monitored, and juveniles were culled to keep densities approximately equal among the four units. Spawned *C. venusta* were removed on 10 September 2012 and then 1 December 2012, euthanized by overdose of MS-222 and preserved in 10% formalin. We only used individuals that had reached adult size in analyses.

### Geometric morphometrics

**Figure 4:**
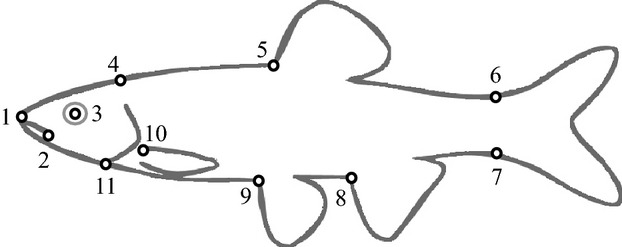
Location of 11 landmarks used to assess body shape variation. The landmarks included: (1) tip of the snout, (2) corner of the mouth, (3) center of the eye, (4) posterior tip of the supraoccipital process, (5) anterior terminus of the dorsal fin base, (6) insertion of the last dorsal ray on the caudal fin, (7) insertion of the last ventral ray on the caudal fin, (8) anterior terminus of the anal fin base, (9) anterior terminus of the pelvic fin base, (10) anterior terminus of the pectoral fin base, and (11) posterior border of the bony opercle and the body outline.

Body shape variation of field-collected and mesocosm-reared specimens was quantified using geometric morphometric analyses (Zelditch et al. 2004) with tps software (http://life.bio.sunysb.edu/morph/) and R (R Development Core Team 2011). The lateral left side of each individual was photographed (Canon PowerShot A1100) with a reference scale, and the order of photographs randomized (to reduce potential biases associated with the sequence specimens were subjected to landmark demarcation), and set 11 homologous landmarks on each photograph using tpsDig2 software (Fig. 4; Rohlf 2004a). We rescaled landmark coordinates using the reference scale, and aligned landmark coordinates using a General Procrustes Analysis (GPA) to remove the effects of scale, translation, and rotation on shape variation for each group separately (i.e., field-collected and mesocosm-reared individuals). Relative warps (hereafter referred to as shape variables) for each group were calculated (*n* = 18) but because some shape variables often do not explain an appreciable amount of variation (Rohlf 1993), we only retained shape variables that explained more than 3% of the variation in shape for each dataset (retained variables explained >89.0% of the variance in each data set). Variation in shape was visualized using thin-plate spline transformation grids in tpsRegr (Rohlf 2004b).

### Data analyses

We developed our analyses to assess the relative contribution of reservoir basin and habitat type in shaping variation from field-collected individuals. We then tested population-level effects and flow-induced phenotypic plasticity on shape variation from individuals reared in mesocosms in flow and non-flow conditions. We then compared and contrasted shape variation between reservoir and stream populations collected from the field to shape variation from mesocosm-reared individuals from reservoir and stream populations reared under flow and non-flow conditions. We predicted *C. venusta* individuals from reservoir habitats would have smaller heads and deeper bodies compared with stream collected individuals and predicted these shape differences between habitats would be conserved in respective flow and non flow-reared individuals. We also predicted individuals reared in flowing conditions would be more streamlined with smaller caudal areas compared with individuals reared in nonflow conditions.

### Field-collected fish

We tested for morphological divergence between stream and reservoir habitats with multivariate analysis of covariance (mancova). All mancova models assume multivariate normality, homogeneity of covariance matrices, independence of observations, linear relationships between covariates and dependent variables, and homogeneity of slopes among groups (Rencher 2002). The mancova model included 8 shape variables (explaining 89.9% of the variation in shape) as dependent variables, standard length (SL) as a covariate (to test for effects of allometry), habitat type (to test for effects of stream or reservoir habitats), basin (to test for basin-level effects) as fixed factors. Heterogeneity of slopes was tested among basins, and between habitat types by including SL in the respective interaction terms. All nonsignificant interaction terms were removed from the final model, and *F*-values were approximated using Wilk's lambda. Because of the statistical power associated with mancova of shape data, we focused our interpretation of model results on effect strengths by use of partial eta squared (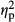) rather than *P*-values. We calculated the relative variance as the partial variance for a given term divided by the maximum partial variance value in the model.

To assess the nature of morphological divergence in reservoir habitats, we calculated a morphological divergence vector as defined by Langerhans (2009) between the two habitat types. This morphological divergence vector does not distort morphological space and summarizes the linear combination of shape variables that contribute to the greatest difference in body shape for a given term of interest (here, reservoir and stream habitats) after controlling for other effects (Langerhans 2009). To quantify this habitat divergence vector, we multiplied the eigenvector of the habitat term's Sums of Squares and Cross Products (SSCP) matrix from the mancova (final model described above) by the shape variables matrix to yield habitat divergence vector scores for each individual. This divergence vector summarizes the shape variation that was elicited in fish from reservoir and stream habitats. The nature of this shape change was visualized using thin-plate spline transformation grids.

### Mesocosm fish

We used mancova to test for population-level differences and flow-induced phenotypic plasticity on body shape variation within mesocosm-reared *C. venusta*. The mancova model included 8 shape variables as dependent variables (explaining 89.0% of the variation in shape), Population (stream or reservoir) and treatment (flow or no flow) were included as fixed factors, and SL as a covariate. Heterogeneity of slopes was tested between populations and treatment by inclusion of SL in each respective interaction term. Nonsignificant interaction terms were omitted from the final model. To quantify the nature of population-level and flow-induced plasticity of body shape variation of mesocosm-reared individuals, divergence vectors for population and treatment were calculated from the final model (similar to above). We visualized shape deformations along each population and treatment divergence vector using thin-plate spline transformation grids. All analyses were conducted in R unless otherwise stated (R Development Core Team 2011).

To investigate the potential contribution of population-level differences and flow-induced phenotypic plasticity of individuals reared in mesocosms to shape divergence observed in the field, we qualitatively compared landmark movements between datasets. We visualized landmark movements along the habitat divergence vector from the field and compared these deformations to landmark movements from population and treatment divergence vectors from mesocosm-reared individuals.

## Results

### Field collections

**Figure 5:**
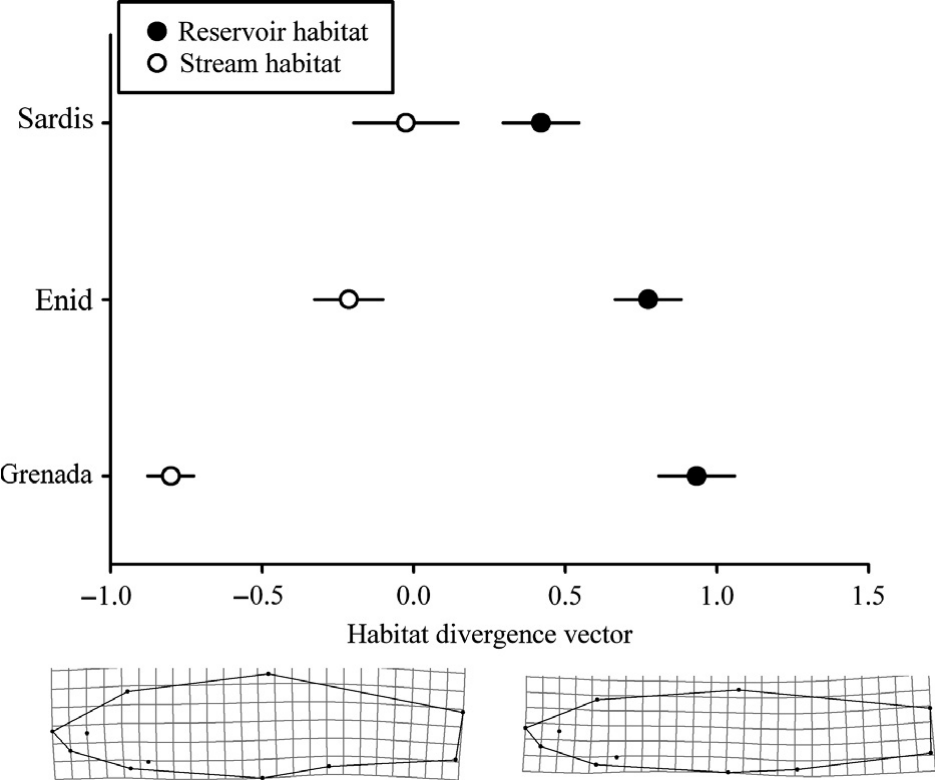
Mean (±1 SE) habitat divergence vector scores for each basin and habitat. Thin-plate spline transformation grids below the axis display the shape transformation between stream and reservoir habitats (magnified 2.5 times to aid in visualization).

When testing for morphological divergence in reservoir habitats, all terms in the global mancova had significant effects on body shape. Standard length had the strongest effect (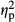 = 0.49), followed by habitat (demonstrating reservoir-induced morphological divergence, 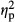 = 0.37), basin (basin-level effects; 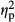 = 0.25), and the Habitat × Basin interaction (showing basin-specific effects; 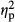 = 0.20). The morphological habitat divergence vector among sites demonstrated consistent divergence between habitat types in the replicate reservoir basins (Fig. 5). In all cases, mean divergence vector scores of reservoir populations were larger than scores of stream populations in each replicate basin. Supporting our prediction, the habitat divergence vector revealed the response to reservoir habitats resulted in an upturn and decreased depth of the head, but contrary to our prediction, reservoir fish showed decreased body depth mainly via ventral movement of the dorsal fin (Fig. 5).

### Mesocosm fish

**Figure 6:**
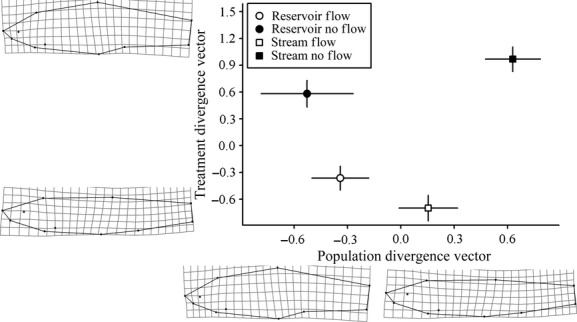
Mean (±1 SE) population and treatment divergence vector scores. Thin-plate spline transformation grids (magnified three times to aid in visualization) on each axis display the shape transformation between populations (*x*-axis) and between flow and nonflow treatments (*y*-axis).

When testing for population-level and flow-induced phenotypic plasticity with mancova, all terms had significant effects on body shape variation (Table 2). Treatment had the strongest effect (indicating flow-induced phenotypic plasticity; 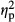 = 0.46), followed by SL (significant allometry; 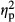 = 0.33), and population (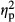 = 0.15). The Population × Treatment interaction also had a significant effect on shape (indicating each population responded differently to the flow treatment; 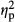 = 0.15). Generally, when exposed to flow conditions offspring from both populations tended to look more similar compared with the offspring reared in nonflow (Fig. 6). Shape deformations related to population-level differences (i.e., shape changes along the population divergence vector) revealed reservoir offspring had smaller relative head sizes and were deeper bodied compared with more fusiform offspring from the stream population. Supporting our prediction, shape variation along the treatment showed fish reared in flow had shallower bodies and larger relative head sizes compared with fish reared in nonflow (Fig. 6).

**Table 2:** mancova results testing for shape divergence in *Cyprinella venusta* from field-collected individuals and offspring reared in stream mesocosms

Model	Effect	Partial variance	Relative variance	df	*F*	*P*
Field collections	SL	0.49	1.00	8,253	30.32	<0.001
	Habitat	0.37	0.76	8,253	18.83	<0.001
	Basin	0.25	0.52	16,506	12.06	<0.001
	Habitat × Basin	0.20	0.41	16,506	8.33	<0.001
Mesocosm fish	Treatment	0.46	1.00	8,95	10.00	<0.001
	SL	0.33	0.72	8,95	5.84	<0.001
	Population	0.15	0.33	8,95	2.14	0.039
	Population × Treatment	0.15	0.33	8,95	2.13	0.040

**Figure 7:**
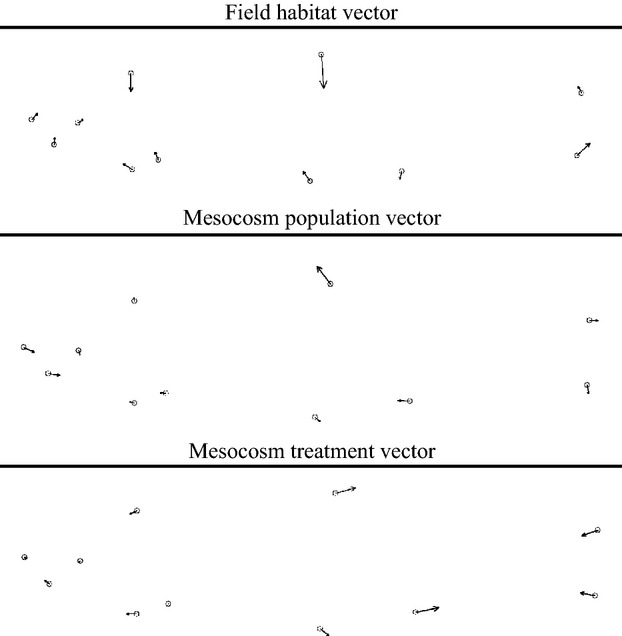
Vector plots showing direction landmarks moved in the field-collected individuals compared with landmark movements based on population-of-origin and flow and nonflow treatments. In both the field habitat vector and mesocosm population vector plots, vectors point in the direction landmarks moved from stream fish to reservoir fish. In the mesocosm treatment vector plot, vectors point in the direction landmarks moved from the flow to nonflow treatment.

Comparing landmark movements from field-collected individuals and fish reared in mesocosms suggested some aspects of reservoir and stream shape were conserved in offspring (Fig. 7). Offspring from reservoir parents retained relatively smaller head sizes compared with offspring from stream parents; however, the shallow body depths of reservoir fish were not retained in mesocosm-reared offspring. Moreover, landmark movements of mesocosm fish when reared in flowing conditions were not concordant with landmark movements between habitat types from field-collected individuals.

## Discussion

We quantified morphological variation of a small-bodied stream fish from reservoir and stream habitats in Mississippi. We also reared offspring from adult individuals collected from a reservoir and stream in flowing and nonflowing stream mesocosms. We found significant and consistent morphological divergence in reservoir habitats. Population-level differences persisted in offspring reared in stream mesocosms, and our data suggested phenotypic plasticity in response to flow in both populations.

Repeated morphological divergence in replicate reservoirs suggests these habitats are facilitating phenotypic changes in populations of *C. venusta*. Individuals from reservoir habitats were more streamlined with smaller heads compared with individuals from stream habitats. However, these morphological changes in the caudal area of *C. venusta* from the field were quite different compared with shape variation of *C. lutrensis* observed in reservoir habitats. Franssen (2011) demonstrated *C. lutrensis* individuals from reservoirs were deeper bodied compared with their stream counterparts. Although some fishes can exhibit more streamlined body shapes in lentic habitats (Hendry et al. 2002; McGuigan et al. 2003; Krabbenhoft et al. 2009), the inconsistency of shape changes between the species was particularly surprising given the relatedness of *C. venusta* and *C. lutrensis*. This disparity in shape changes between the two species in reservoir habitats could be due to different selective pressures between sets of reservoirs from different regions (i.e., between reservoirs in Oklahoma and Mississippi). We predict these species-specific responses are more likely due to underlying genetic variation and ecologies between the species that are interacting with similar environmental conditions in reservoirs.

Morphological diversification in fishes has been linked to dissolved oxygen concentrations and light availability (e.g., Chapman et al. 2000; Langerhans et al. 2007; Witte et al. 2008), flow regimes (e.g., Walker 1997, Hendry et al. 2006, Langerhans 2008), and predator densities (e.g., Domenici and Blake 1997, Langerhans and DeWitt 2004; Hendry et al. 2006, Langerhans et al. 2009). Conversion of natural stream reaches into lentic reservoirs likely alters multiple biotic and abiotic environmental conditions (e.g., turbidity, flow variation, temperature, biotic communities). How fishes respond to these altered conditions will likely depend on their evolutionary histories and species-specific ecologies. Although distantly related fishes can demonstrate similar morphological changes likely linked to swimming performance in reservoir habitats, they also can show species-specific responses (Franssen et al. 2013). Innate differences in the behavioral or trophic ecologies of *C. venusta* and *C. lutrensis* may explain their disparate responses to reservoir habitats, although data on their ecologies are limited. A better understanding the ecologies of these species or differences in their underlying genetic architecture may help elucidate mechanisms behind their disparate morphological changes in reservoir habitats.

Morphological divergence in reservoirs may confer greater fitness to reservoir-resident individuals, facilitating local adaptation in these habitats. Investigations of phenotypic variation in other fishes between lake-stream pairs suggest local habitats can drive phenotypic variation in spite of close proximities of populations (Brinsmead and Fox 2002, Hendry et al. 2002; Berner et al. 2009). However, high migration rates among reservoir and stream populations would limit local adaptation in reservoirs. We know of no physical barriers (e.g., dams or impassible falls) between our reservoir and stream sites that would limit movement of individuals between the habitat types. Yet, the novel environmental conditions of reservoirs may limit movement of small-bodied fishes through reservoir habitats, especially by fluvial specialists (Skalksi et al. 2008; Franssen 2012, Hudman and Gido 2013). With new improvements in molecular techniques (e.g., next generation sequencing), genetic variation responsible for morphological divergence in reservoirs and effects of migration among habitats could be elucidated.

Shape differences in reservoir and stream offspring reared with and without flowing water suggest both genotypic variation and phenotypic plasticity contributed to phenotypic differentiation. While we were unable to estimate heritability, population-level differences persisted in offspring, but morphological similarities between field and mesocosm-reared fish were limited. When reared in mesocosms, differences in the caudal region of fish from the reservoir and stream habitats were not conserved and were qualitatively reversed (i.e., offspring from the reservoir habitat more resembled phenotypic variation of field-collected stream fish). This reversal of caudal regions of the two populations in mesocosms compared with field-collected fish likely indicates fish in the field are exposed to plasticity-inducing factors that were absent in mesocosms. In fishes, there is a general propensity for species inhabiting moving water to be more streamlined than fishes in lentic habitats (Langerhans 2008). Therefore, there are likely strong genetic-environmental interactions shaping phenotypic variation of individuals in stream and reservoir habitats that overcome the genetically based tendency for stream *C. venusta* to be more streamlined than reservoir *C. venusta*. However, relatively smaller head sizes of reservoir fish persisted when individuals were reared in mesocosms. The differences in caudal morphologies of the two populations reared in mesocosms may suggest head morphology may be under stronger selection than caudal body shape in reservoir habitats. In addition, the reversal of caudal shapes of fish between the two populations reared in mesocosms coupled with strong flow-induced changes to caudal regions indicates the caudal areas are likely more plastic compared with anterior regions of the body.

Flow-induced plasticity had the strongest effect on body shape in mesocosm-reared fish, but the significant interaction between treatment and population indicates that populations responded differently to water flow. Yet the plastic shift in morphology by both populations increased their phenotypic similarity rather than their dissimilarity. The phenotypic changes associated with flow did not match variation between habitat types in the field (i.e., reservoir individuals in the field were shallower bodied compared with fish from streams while offspring from a reservoir and a stream habitat reared in nonflow conditions were both deeper bodied). While continued exposure to environmental cues that elicit plasticity of traits can result in canalization (Waddington 1942; Schmalhausen 1949; Debat and David 2001), flow-induced phenotypic plasticity was likely not responsible for phenotypic divergence between habitat types in the field. Phenotypic plasticity along other environmental gradients between stream and reservoir habitats may contribute to observed phenotypic variation in the field, making these traits potentially susceptible to canalization in reservoir habitats.

Lack of basin-level replication in the mesocosm experiment may limit our ability to extrapolate our results and interpretations to other reservoir systems. This would be especially true if drift, mutation, or recombination had stronger effects than selection on the genetic structure of *C. venusta* in the Grenada basin. We suggest this is an unlikely scenario given the apparently large population sizes of *C. venusta* in all the habitats we investigated (i.e., *C. venusta* was very common). Nonetheless, the mancova of body shape variation of field-collected individuals indicated Basin and the Basin × Habitat interaction had significant effects on body shape variation, indicating fish body shape varied among basins and had dissimilar responses to reservoir habitats among basins. However, both of these effects explained less variation than the habitat term, suggesting variation between habitat types had a stronger influence on body shape variation than variation due to genetic variation among basins. While the lack of basin-level replication in the mesocosm experiment was not ideal, we think it is unlikely and have no evidence that would suggest nonadaptive evolutionary processes shaped the genetic structure of *C. venusta* in the Grenada basin.

The inability to elicit similar phenotypic variation in individuals reared in flow variation (this paper) and in the presence of predators (Franssen 2011) to morphological variation observed from field-collected individuals suggests divergent morphological variation in reservoirs is not due to flow- or predator-induced plasticity. Moreover, the unexpected phenotypic variation of *C. venusta* in reservoir habitats (compared with *C. lutrensis*) indicates selective pressures may vary among reservoirs or that different species respond to similar selective pressures in different fashions. Together, investigations of morphological changes of fishes in reservoir habitats may suggest that the reduction in factors that can contribute to morphological variation to one or two variables (e.g., flow variation or predator densities) may be an over-simplification when comparing phenotypic variation in different habitats. Indeed, a multitude of environmental conditions likely covary between these different habitats. A better understanding of the spatial and temporal variation in other potential environmental selective pressures and how these conditions interact with genetic variation to produce phenotypic variation will be needed to understand how reservoirs can alter the evolutionary trajectories of resident populations.

Most organisms live in environments that have been altered at least to some extent by humans (Palumbi 2001). It will likely be difficult to quantify how complex temporal and spatial scale dependent environmental change may present organisms with evolutionary novel selective pressures (Sih et al. 2011). Furthermore, unique evolutionary histories and ecologies of species make predicting rapid evolutionary responses to human-modified habitats difficult (Sih et al. 2011). A major challenge in the coming decades will be to understand how human-induced evolutionary change will shape traits of organisms and the influence of trait changes on larger ecological processes (Palkovacs et al. 2011).
